# (*S*)-2-[(2-Ammonio­phenyl)­sulfanyl­methyl]pyrrolidinium dibromide

**DOI:** 10.1107/S1600536808021089

**Published:** 2008-07-19

**Authors:** Bailin Li, Shuai Zhang, Yifeng Wang, Shuping Luo

**Affiliations:** a State Key Laboratory Breeding Base of Green Chemistry-Synthesis Technology, Zhejiang University of Technology, Hangzhou 310014, People’s Republic of China; b Department of Pharmaceutical and Chemical Engineering, Taizhou College, Linhai, Zhejiang 317000, People’s Republic of China

## Abstract

In the title compound, C_11_H_18_N_2_S^2+^·2Br^−^, the pyrrolidine ring displays a half-chair conformation, with the flap C atom lying 0.522 (5) Å out of the plane of the other four atoms. The methyl­ene C atom, which connects the pyrrolidinium ring and the thio­ether group, is displaced from the plane of four pyrrolidinium atoms by 0.690 (6) Å in the same direction as the flap C atom. The plane of four pyrrolidinium atoms is almost perpendicular to the benzene ring [dihedral angle = 75.02 (4)°]. The crystal structure is stabilized by hydrogen bonds between the N and Br atoms.

## Related literature

The synthesis of (*S*)-(+)-2-bromo­methyl­pyrrolidine hydro­bromide was described by Xu *et al.* (2006 ▶). The development of asymmetric organocatalysis was reviewed by Seayad & List (2005 ▶).
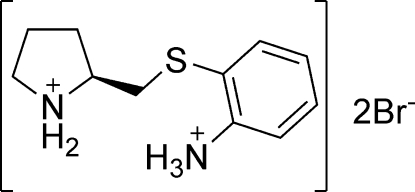


## Experimental

### 

#### Crystal data

C_11_H_18_N_2_S^2+^·2Br^−^
*M* *_r_* = 370.15
Orthorhombic, 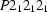

*a* = 7.9399 (9) Å
*b* = 10.8427 (13) Å
*c* = 17.658 (2) Å
*V* = 1520.2 (3) Å^3^
*Z* = 4
Mo *K*α radiation
μ = 5.45 mm^−1^
*T* = 293 (2) K
0.49 × 0.42 × 0.36 mm

#### Data collection

Bruker SMART CCD area-detector diffractometer
Absorption correction: multi-scan (*SADABS*; Sheldrick, 1996 ▶) *T*_min_ = 0.103, *T*_max_ = 0.137
8969 measured reflections
3311 independent reflections
1808 reflections with *I* > 2σ(*I*)
*R*_int_ = 0.136

#### Refinement

*R*[*F* ^2^ > 2σ(*F* ^2^)] = 0.058
*wR*(*F* ^2^) = 0.134
*S* = 0.83
3311 reflections
158 parameters
3 restraints
H atoms treated by a mixture of independent and constrained refinement
Δρ_max_ = 0.67 e Å^−3^
Δρ_min_ = −0.50 e Å^−3^
Absolute structure: Flack (1983 ▶), 1394 Friedel pairs
Flack parameter: 0.00 (2)

### 

Data collection: *SMART* (Bruker, 2001 ▶); cell refinement: *SAINT-Plus* (Bruker, 2000 ▶); data reduction: *SAINT-Plus*; program(s) used to solve structure: *SHELXS97* (Sheldrick, 2008 ▶); program(s) used to refine structure: *SHELXL97* (Sheldrick, 2008 ▶); molecular graphics: *SHELXTL* (Sheldrick, 2008 ▶); software used to prepare material for publication: *SHELXTL*.

## Supplementary Material

Crystal structure: contains datablocks global, I. DOI: 10.1107/S1600536808021089/pk2105sup1.cif

Structure factors: contains datablocks I. DOI: 10.1107/S1600536808021089/pk2105Isup2.hkl

Additional supplementary materials:  crystallographic information; 3D view; checkCIF report

## Figures and Tables

**Table 1:** Hydrogen-bond geometry (Å, °)

*D*—H⋯*A*	*D*—H	H⋯*A*	*D*⋯*A*	*D*—H⋯*A*
N2—H2*E*⋯Br1^i^	0.83 (8)	2.39 (8)	3.201 (9)	169 (10)
N2—H2*D*⋯Br2^ii^	0.84 (6)	2.48 (4)	3.277 (9)	159 (8)
N2—H2*C*⋯Br1	0.84 (7)	2.47 (7)	3.298 (9)	173 (8)
N1—H1*B*⋯Br2	0.90	2.47	3.355 (7)	169
N1—H1*A*⋯Br2^ii^	0.90	2.33	3.224 (7)	170

Symmetry codes: (i) 
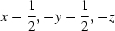
; (ii) 
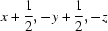
.

**Fig. 1.**
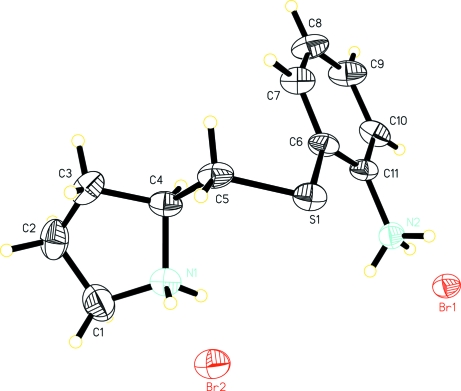
The asymmetric unit of the title compound with the atomic labeling scheme. Displacement ellipsoids are drawn at the 50% probability level.

**Fig. 2.**
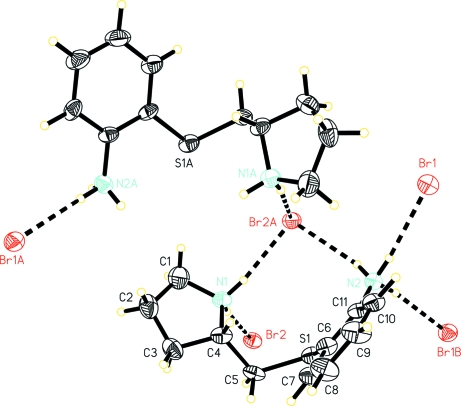
The molecular packing of the title compound showing H-bridge interactions between cationic-anionic groups.

## supplementary crystallographic information

### Comment

In recent years, the field of asymmetric organocatalysis has developed rapidly, attracting an increasing number of research groups around the world (Seayad & List, 2005). The title compound, readily synthesized from commercially available *L*-proline and 2-aminobenzenethiol, could act as an organocatalyst in the Michael addition of ketones to nitrostyrenes. The reaction gave the desired Michael adducts in good yields and high enantioselectivities. The structure of (*S*)-2-((2-ammoniophenylthio)methyl)pyrrolidinium dibromide is shown in Fig. 1.

The crystal is built of doubly protonated cations and bromide anions. The pyrrolidine ring displays a half-chair conformation, with the flap C atom lying 0.522 (5) Å from the remaining four atoms of the pyrrolidine which are almost coplanar. The methylene C atom, which connects the pyrrolidinium ring and the thioether group, is displaced from the plane of four pyrrolidinium atoms by 0.690 (6) Å in the same direction, as the flap C atom. The plane of four pyrrolidinium ring atoms is almost perpendicular to the benzene ring [dihedral angle 75.02 (4) °]. The crystal structure is stabilized by hydrogen-bonds between the N and Br atoms. The molecular packing of the title compound showing H-bridge interactions between cationic-anionic groups is shown in Fig. 2.

### Experimental

The title compound was synthesized by treating 2-aminobenzenethiol (1.25 g,10 mmol) with (*S*)-2-bromomethylpyrrolidine hydrobromide (2.47 g,10 mmol) in MeCN (30 ml) under stirring at 353 K for 24 h (yield 87%). The compound (*S*)-2-bromomethylpyrrolidine hydrobromide was obtained from commercially available *L*-proline by reduction with NaBH_4_ and subsequent bromination with PBr_3_ (Xu *et al.*, 2006). Suitable crystals of the title compound were obtained by slow evaporation of an ethanol solution at room temperature.

### Refinement

All carbon-bonded H atoms were placed in calculated positions with C—H = 0.93 Å (C_ar_), C—H = 0.98 Å (R_3_CH), C—H = 0.97 Å (R_2_CH_2_) and refined using a riding model, with *U*_iso_(H)=1.2_eq_(C). NH_3_ hydrogen atoms were located in a difference map and refined with an N—H distance restraint of 0.83 (1) Å, with U value being 0.06, 0.06, 0.09 respectively, while NH_2_ hydrogens were treated using a riding model with N—H distance of 0.90 Å.

### Crystal data

**Table:**

C_11_H_18_N_2_S^2+^·2Br^–^	*F*_000_ = 736
*M**_r_* = 370.15	*D*_x_ = 1.617 Mg m^−^^3^
Orthorhombic, *P*2_1_2_1_2_1_	Mo *K*α radiation λ = 0.71073 Å
Hall symbol: P 2ac 2ab	Cell parameters from 1525 reflections
*a* = 7.9399 (9) Å	θ = 4.4–38.3º
*b* = 10.8427 (13) Å	µ = 5.45 mm^−^^1^
*c* = 17.658 (2) Å	*T* = 293 (2) K
*V* = 1520.2 (3) Å^3^	Prismatic, colorless
*Z* = 4	0.49 × 0.42 × 0.37 mm

### Data collection

**Table:**

Bruker SMART CCD area-detector diffractometer	3311 independent reflections
Radiation source: fine-focus sealed tube	1808 reflections with *I* > 2σ(*I*)
Monochromator: graphite	*R*_int_ = 0.136
*T* = 293(2) K	θ_max_ = 27.0º
φ and ω scans	θ_min_ = 2.2º
Absorption correction: multi-scan(SADABS; Sheldrick, 1996)	*h* = −9→10
*T*_min_ = 0.103, *T*_max_ = 0.137	*k* = −13→12
8969 measured reflections	*l* = −22→18

### Refinement

**Table:**

Refinement on *F*^2^	Hydrogen site location: inferred from neighbouring sites
Least-squares matrix: full	H atoms treated by a mixture of independent and constrained refinement
*R*[*F*^2^ > 2σ(*F*^2^)] = 0.058	*w* = 1/[σ^2^(*F*_o_^2^) + (0.049*P*)^2^] where *P* = (*F*_o_^2^ + 2*F*_c_^2^)/3
*wR*(*F*^2^) = 0.134	(Δ/σ)_max_ < 0.001
*S* = 0.83	Δρ_max_ = 0.67 e Å^−^^3^
3311 reflections	Δρ_min_ = −0.50 e Å^−^^3^
158 parameters	Extinction correction: SHELXL97 (Sheldrick, 2008), Fc^*^=kFc[1+0.001xFc^2^λ^3^/sin(2θ)]^-1/4^
3 restraints	Extinction coefficient: 0.0005 (1)
Primary atom site location: structure-invariant direct methods	Absolute structure: Flack (1983), 1394 Friedel pairs
Secondary atom site location: difference Fourier map	Flack parameter: 0.00 (2)

### Special details

**Table:**

Geometry. All e.s.d.'s (except the e.s.d. in the dihedral angle between two l.s. planes) are estimated using the full covariance matrix. The cell e.s.d.'s are taken into account individually in the estimation of e.s.d.'s in distances, angles and torsion angles; correlations between e.s.d.'s in cell parameters are only used when they are defined by crystal symmetry. An approximate (isotropic) treatment of cell e.s.d.'s is used for estimating e.s.d.'s involving l.s. planes.
Refinement. Refinement of *F*^2^ against ALL reflections. The weighted *R*-factor *wR* and goodness of fit *S* are based on *F*^2^, conventional *R*-factors *R* are based on *F*, with *F* set to zero for negative *F*^2^. The threshold expression of *F*^2^ > 2σ(*F*^2^) is used only for calculating *R*-factors(gt) *etc*. and is not relevant to the choice of reflections for refinement. *R*-factors based on *F*^2^ are statistically about twice as large as those based on *F*, and *R*- factors based on ALL data will be even larger.

### Fractional atomic coordinates and isotropic or equivalent isotropic displacement parameters (Å^2^)

**Table:**

	*x*	*y*	*z*	*U*_iso_*/*U*_eq_	
Br1	0.30470 (12)	−0.24305 (9)	−0.08797 (5)	0.0559 (3)	
Br2	−0.38177 (12)	0.34415 (8)	0.06967 (5)	0.0526 (3)	
S1	−0.0443 (3)	0.1034 (2)	0.14946 (13)	0.0524 (6)	
N1	0.0399 (9)	0.3571 (6)	0.0600 (4)	0.0502 (18)	
H1A	0.0636	0.2939	0.0288	0.060*	
H1B	−0.0728	0.3650	0.0630	0.060*	
N2	0.1147 (12)	−0.0815 (8)	0.0452 (5)	0.0487 (18)	
C1	0.1159 (18)	0.4726 (9)	0.0308 (7)	0.083 (3)	
H1C	0.0296	0.5273	0.0111	0.100*	
H1D	0.1959	0.4549	−0.0093	0.100*	
C2	0.2025 (14)	0.5297 (9)	0.0973 (7)	0.072 (3)	
H2A	0.3237	0.5225	0.0921	0.087*	
H2B	0.1735	0.6164	0.1013	0.087*	
C3	0.1443 (14)	0.4625 (9)	0.1640 (6)	0.068 (3)	
H3A	0.0420	0.4990	0.1840	0.081*	
H3B	0.2297	0.4627	0.2033	0.081*	
C4	0.1120 (12)	0.3339 (8)	0.1362 (4)	0.049 (2)	
H4	0.2194	0.2901	0.1309	0.059*	
C5	−0.0079 (11)	0.2577 (8)	0.1852 (4)	0.052 (2)	
H5A	−0.1150	0.3004	0.1885	0.062*	
H5B	0.0381	0.2519	0.2359	0.062*	
C6	0.1553 (11)	0.0316 (8)	0.1640 (5)	0.047 (2)	
C7	0.2504 (11)	0.0537 (9)	0.2270 (5)	0.056 (3)	
H7	0.2117	0.1092	0.2632	0.068*	
C8	0.4032 (13)	−0.0049 (10)	0.2382 (6)	0.073 (3)	
H8	0.4680	0.0138	0.2805	0.087*	
C9	0.4579 (14)	−0.0887 (10)	0.1879 (6)	0.071 (3)	
H9	0.5602	−0.1285	0.1956	0.085*	
C10	0.3611 (11)	−0.1161 (8)	0.1239 (5)	0.053 (2)	
H10	0.3980	−0.1750	0.0893	0.063*	
C11	0.2112 (12)	−0.0555 (8)	0.1123 (5)	0.045 (2)	
H2C	0.157 (10)	−0.127 (6)	0.012 (4)	0.06 (3)*	
H2D	0.094 (12)	−0.032 (6)	0.010 (3)	0.06 (3)*	
H2E	0.043 (10)	−0.135 (7)	0.054 (6)	0.09 (4)*	

### Atomic displacement parameters (Å^2^)

**Table:**

	*U*^11^	*U*^22^	*U*^33^	*U*^12^	*U*^13^	*U*^23^
Br1	0.0514 (6)	0.0627 (5)	0.0536 (6)	0.0081 (5)	−0.0055 (4)	−0.0024 (5)
Br2	0.0526 (6)	0.0614 (5)	0.0438 (5)	0.0072 (5)	−0.0041 (4)	−0.0022 (5)
S1	0.0412 (14)	0.0682 (15)	0.0479 (14)	0.0052 (12)	0.0015 (11)	0.0049 (12)
N1	0.045 (4)	0.060 (4)	0.046 (4)	0.007 (4)	0.004 (3)	−0.001 (4)
N2	0.054 (5)	0.048 (5)	0.044 (5)	−0.003 (5)	0.002 (5)	−0.002 (4)
C1	0.105 (10)	0.061 (7)	0.084 (8)	0.010 (7)	0.005 (8)	0.006 (6)
C2	0.055 (7)	0.055 (6)	0.107 (10)	0.005 (5)	0.010 (7)	−0.017 (6)
C3	0.062 (8)	0.074 (7)	0.067 (7)	−0.006 (6)	−0.007 (6)	−0.021 (6)
C4	0.038 (5)	0.066 (6)	0.044 (5)	0.015 (5)	0.000 (4)	0.003 (5)
C5	0.053 (6)	0.070 (6)	0.033 (4)	0.019 (6)	0.002 (4)	−0.009 (5)
C6	0.033 (6)	0.062 (6)	0.046 (5)	0.008 (4)	−0.006 (4)	0.010 (4)
C7	0.043 (6)	0.077 (7)	0.048 (6)	0.012 (5)	0.003 (5)	0.000 (5)
C8	0.062 (8)	0.102 (8)	0.055 (7)	0.010 (7)	−0.022 (6)	−0.008 (6)
C9	0.054 (7)	0.103 (8)	0.056 (7)	0.028 (7)	−0.005 (6)	−0.001 (6)
C10	0.046 (6)	0.061 (6)	0.051 (5)	0.022 (5)	0.011 (5)	0.010 (4)
C11	0.044 (6)	0.059 (6)	0.031 (5)	0.000 (5)	0.000 (4)	0.007 (4)

### Geometric parameters (Å, °)

**Table:**

Br1—H2C	2.47 (7)	C3—C4	1.500 (12)
Br2—H1B	2.4665	C3—H3A	0.9700
S1—C6	1.784 (8)	C3—H3B	0.9700
S1—C5	1.811 (9)	C4—C5	1.529 (12)
N1—C1	1.483 (12)	C4—H4	0.9800
N1—C4	1.484 (10)	C5—H5A	0.9700
N1—H1A	0.9000	C5—H5B	0.9700
N1—H1B	0.9000	C6—C7	1.366 (11)
N2—C11	1.438 (11)	C6—C11	1.388 (11)
N2—H2C	0.84 (7)	C7—C8	1.383 (13)
N2—H2D	0.84 (6)	C7—H7	0.9300
N2—H2E	0.83 (8)	C8—C9	1.343 (13)
C1—C2	1.495 (14)	C8—H8	0.9300
C1—H1C	0.9700	C9—C10	1.399 (13)
C1—H1D	0.9700	C9—H9	0.9300
C2—C3	1.460 (13)	C10—C11	1.374 (12)
C2—H2A	0.9700	C10—H10	0.9300
C2—H2B	0.9700		
			
C6—S1—C5	102.2 (4)	N1—C4—C3	101.8 (7)
C1—N1—C4	107.5 (8)	N1—C4—C5	111.3 (7)
C1—N1—H1A	110.2	C3—C4—C5	115.1 (8)
C4—N1—H1A	110.2	N1—C4—H4	109.4
C1—N1—H1B	110.2	C3—C4—H4	109.4
C4—N1—H1B	110.2	C5—C4—H4	109.4
H1A—N1—H1B	108.5	C4—C5—S1	113.7 (6)
C11—N2—H2C	118 (6)	C4—C5—H5A	108.8
C11—N2—H2D	126 (6)	S1—C5—H5A	108.8
H2C—N2—H2D	87 (7)	C4—C5—H5B	108.8
C11—N2—H2E	111 (7)	S1—C5—H5B	108.8
H2C—N2—H2E	90 (9)	H5A—C5—H5B	107.7
H2D—N2—H2E	117 (10)	C7—C6—C11	118.6 (8)
N1—C1—C2	105.3 (9)	C7—C6—S1	122.1 (7)
N1—C1—H1C	110.7	C11—C6—S1	119.1 (7)
C2—C1—H1C	110.7	C6—C7—C8	121.3 (9)
N1—C1—H1D	110.7	C6—C7—H7	119.3
C2—C1—H1D	110.7	C8—C7—H7	119.3
H1C—C1—H1D	108.8	C9—C8—C7	120.0 (10)
C3—C2—C1	106.3 (8)	C9—C8—H8	120.0
C3—C2—H2A	110.5	C7—C8—H8	120.0
C1—C2—H2A	110.5	C8—C9—C10	120.1 (9)
C3—C2—H2B	110.5	C8—C9—H9	120.0
C1—C2—H2B	110.5	C10—C9—H9	120.0
H2A—C2—H2B	108.7	C11—C10—C9	119.6 (9)
C2—C3—C4	104.7 (7)	C11—C10—H10	120.2
C2—C3—H3A	110.8	C9—C10—H10	120.2
C4—C3—H3A	110.8	C10—C11—C6	120.3 (8)
C2—C3—H3B	110.8	C10—C11—N2	119.4 (8)
C4—C3—H3B	110.8	C6—C11—N2	120.3 (8)
H3A—C3—H3B	108.9		
			
C4—N1—C1—C2	−12.2 (11)	C11—C6—C7—C8	2.8 (14)
N1—C1—C2—C3	−12.1 (11)	S1—C6—C7—C8	178.6 (8)
C1—C2—C3—C4	31.4 (11)	C6—C7—C8—C9	−2.4 (15)
C1—N1—C4—C3	30.7 (10)	C7—C8—C9—C10	0.5 (16)
C1—N1—C4—C5	153.8 (8)	C8—C9—C10—C11	1.0 (15)
C2—C3—C4—N1	−38.0 (10)	C9—C10—C11—C6	−0.6 (13)
C2—C3—C4—C5	−158.6 (8)	C9—C10—C11—N2	178.2 (9)
N1—C4—C5—S1	64.5 (8)	C7—C6—C11—C10	−1.3 (12)
C3—C4—C5—S1	179.7 (7)	S1—C6—C11—C10	−177.2 (6)
C6—S1—C5—C4	69.0 (6)	C7—C6—C11—N2	180.0 (8)
C5—S1—C6—C7	38.7 (8)	S1—C6—C11—N2	4.1 (11)
C5—S1—C6—C11	−145.6 (7)		

### Hydrogen-bond geometry (Å, °)

**Table:**

*D*—H···*A*	*D*—H	H···*A*	*D*···*A*	*D*—H···*A*
N2—H2E···Br1^i^	0.83 (8)	2.39 (8)	3.201 (9)	169 (10)
N2—H2D···Br2^ii^	0.84 (6)	2.48 (4)	3.277 (9)	159 (8)
N2—H2C···Br1	0.84 (7)	2.47 (7)	3.298 (9)	173 (8)
N1—H1B···Br2	0.90	2.47	3.355 (7)	169
N1—H1A···Br2^ii^	0.90	2.33	3.224 (7)	170

Symmetry codes: (i) *x*−1/2, −*y*−1/2, −*z*; (ii) *x*+1/2, −*y*+1/2, −*z*.

## References

[bb1] Bruker (2000). *SAINT-Plus* Bruker AXS Inc., Madison, Wisconsin, USA.

[bb2] Bruker (2001). *SMART* Bruker AXS Inc., Madison, Wisconsin, USA.

[bb3] Flack, H. D. (1983). *Acta Cryst.* A**39**, 876–881.

[bb4] Seayad, J. & List, B. (2005). *Org. Biol. Chem.* **3**, 719–724. 10.1039/b415217b 15731852

[bb5] Sheldrick, G. M. (1996). *SADABS* University of Göttingen, Germany.

[bb6] Sheldrick, G. M. (2008). *Acta Cryst.* A**64**, 112–122. 10.1107/S0108767307043930 18156677

[bb7] Xu, D. Q., Luo, S. P., Yue, H. D., Wang, L. P., Liu, Y. K. & Xu, Z. Y. (2006). *Synlett*, **16**, 2569–2572.

